# Distal acupoint stimulation versus peri-incisional stimulation for postoperative pain in open abdominal surgery: a systematic review and implications for clinical practice

**DOI:** 10.1186/s12906-019-2583-8

**Published:** 2019-07-30

**Authors:** Juan Zhu, Qian Xu, Rong Zou, Wenzhong Wu, Xiaoqiu Wang, Yanyi Wang, Fangbing Ji, Zhen Zheng, Man Zheng

**Affiliations:** 1 Department of Anesthesiology, Affiliated Hospital of Nanjing University of Traditional Chinese Medicine, Hanzhong Road 155, Nanjing, 210029 China; 2 Department of Acupuncture and Rehabilitation, Affiliated Hospital of Nanjing University of Chinese Medicine, Hanzhong Road 155, Nanjing, 210029 China; 3 Discipline of Chinese Medicine, School of Health and Biomedical Sciences, RMIT University, PO BOX 71, Bundoora, VIC 3083 Australia

**Keywords:** Distal acupoint stimulation, Peri-incisional stimulation, Postoperative pain, Open abdominal surgery

## Abstract

**Background:**

Acute postoperative pain remains a major clinical problem that affects patient recovery. Distal acupoint and peri-incisional stimulation are both used for relieving acute postoperative pain in hospital. Our objective was to assess and compare the effects of distal and peri-incisional stimulation on postoperative pain in open abdominal surgery.

**Methods:**

MEDLINE, EMBASE, Cochrane Central Register of Controlled Trials and Chinese databases CNKI and Wanfangdata were searched to identify eligible randomized controlled trials. Intensity of postoperative pain, opioid consumption and related data were extracted and analyzed using a random effects model. Risk of bias was assessed. Subgroup analyses were conducted when data were enough.

**Results:**

Thirty-five trials were included, in which 17 trials studied distal stimulation, another 17 trials studied peri-incisional stimulation and one studied the combination of the two approaches. No studies that directly compared the two approaches were identified. Subgroup analysis showed that both distal and peri-incisional stimulation significantly alleviated postoperative resting and movement pain from 4 h to 48 h after surgery by 6 to 25 mm on a 100 mm visual analogue scale. Peri-incisional stimulation showed a better reduction in postoperative opioid consumption. No studies compared the effects of the combined peri-incisional and distal stimulation with either mode alone. Overall the quality of evidence was moderate due to a lack of blinding in some studies, and unclear risk of allocation concealment.

**Conclusion:**

Both distal and peri-incisional modes of stimulation were effective in reducing postoperative pain. Whether a combined peri-incisional stimulation and distal acupuncture has superior results requires further studies.

## Background

Abdominal surgery is one of most common types of surgery and takes up to 50% surgery expenditure [1]. It is also one of the most painful types of surgery [2], and more than 80% of these patients suffer from moderate to severe postoperative pain[3, 4]. Severe abdominal pain significantly impacts on patient recovery and quality of life [5]. Providing effective pain relief in this group of patients is a major challenge for surgeons and anesthetists. Multimodal analgesic strategies of combined opioids and non-opioids drugs are the standard management, but cannot fully relieve pain and are associated with many adverse effects, such as nausea, vomiting, dizziness, and constipation [6]. Such adverse effects compound the common postoperative complications associated with abdominal surgery. To improve postoperative pain management, alternative, non-pharmacological therapies with minimal adverse effects on the gut are needed [7].

Acupuncture is one of the most common non-pharmacological therapies for pain [8, 9]. Strong evidence demonstrates that acupuncture treatment is effective for acute dental pain and postoperative nausea and vomiting [10, 11]. Some studies show that acupuncture has opioid-sparing effects making it a useful alternative or adjunct therapy to conventional management of postoperative pain. For managing postoperative pain, some studies stimulated acupuncture points (acupoints) on the arms and legs away from the pain sites, whereas other studies stimulated points at or close to the site of pain [12, 13]. Each has its advantages and disadvantages in clinical practice. It is unknown which mode of stimulation is better or if a combined peri-incisional and distal stimulation is better than alone. Acupoint stimulation, mostly used in the form of needling or acupressure, has been increasingly used to alleviate postoperative pain. A systematic review based on 15 studies and 1166 participants evaluated the effects of acupoint stimulation on postoperative pain, and found that acupuncture could significantly and safely reduce postoperative pain and reduce opioid consumption when compared with sham acupuncture [12].

Peri-incisional stimulation applies needles or uses a TENS (Transcutaneous electrical nerve stimulation) or TENS like machine to deliver alternating current via cutaneous electrodes positioned near the painful site. There have been reviews of TENS on management of postoperative pain, and presented different results [14]. A systematic review of 11 studies with positive effects showed that adequate stimulation parameters could significantly reduce postoperative analgesic intake [15]. The other review showed however that TENS might reduce movement pain, but not postoperative resting pain [16].

The aims of this systematic review were 1) to compare the effects of peri-incisional stimulation with distal acupoint stimulation in treating postoperative pain; 2) to assess if combined distal acupuncture and peri-incisional stimulation was better than either alone.

## Methods

This review adheres to the PRISMA guidance [17]. Randomized controlled clinical trials that studied the effects of distal stimulation or stimulation at the incision site, on postoperative pain in patients undergoing open abdominal surgery were searched in databases of MEDLINE, EMBASE, and Cochrane Central Register of Controlled Trials (CENTRAL). Chinese trials were searched in two Chinese Databases, CNKI and wanfangdata. The last electronic search was in September 2016.

### Selection criteria

#### Inclusion criteria

To be included, studies must have met the following criteria: 1. randomized controlled clinical trials (RCTs); 2. patients underwent open abdominal surgery regardless of age, gender, ethnicity, type of anesthesia; 3. all forms of acupuncture and TENS or peri-incisional stimulation was used; 4. full text articles in English or Chinese; 5. must have reported postoperative pain (Mean and SD) or analgesic use (mean and SD) within 24 to 48 h postoperatively; 6. Control group consisted of no stimulation, sham stimulation, other forms of stimulation.

#### Exclusion criteria were

1. patients with other co-existing acute or chronic illness; 2. Laparoscopic surgery 3. duplicate articles; 4. stimulation on cutaneous nerves but not points close to the defined incision site; 5. articles only reported incidence of pain relief which needed opioid treatment, but without reporting the intensity of pain or dosage of opioid consumption.

### Outcome measures

Primary outcomes: 1. Postoperative pain including resting pain and pain on movement or cough at 6, 12, 24, or 48 h. 2. Postoperative opioid usage.

Secondary outcomes: 1. Adverse events of opioids.2. Anaesthetics usage. 3. Extubation time. 4. Days in hospital. 5. Duration in PACU (post-anesthesia care unit). 6. Return to activity.

### Data collection

Two reviewers (Z.J and Z.R) independently screened the search results, selected studies, extracted data and assessed the risk of bias using a data extraction Excel file. The third reviewer (Z.Z) was consulted when disagreements occurred until a consensus was achieved. Then, data were checked by another reviewer (FB.J). The STRICATA (Standards for Reporting Interventions in Controlled Trials of Acupuncture) was extracted by XQ.W and WZ.W who both majored in acupuncture.

The authors were contacted if the data were insufficient. The data were not used in the review if no response was received from correspondence authors. In studies with more than two groups, we avoided double-counting of participants by following the guidelines for selecting studies and collecting data in the *Cochrane Handbook for Systematic Reviews of Interventions* [12]. The following data were extracted: (i) types of stimulation, (ii) type of surgery, (iii) type of anesthesia, (iv) comparison groups, subgroups and number of patients, (v) pain scores including resting pain and pain on movement or cough at 4, 12, 24, 48 and 72 h after operation, (vi) total opioid analgesic consumption at 24, 48 and 72 h after operation, (vii) the incidence of opioid-related side-effects, such as vomiting, nausea, dizziness and pruritus, and (viii) anaesthetics usage and duration of recovery room stay. Pain scores were recorded and analyzed as visual analogue pain scores (VAS, 0–100 mm). Verbal rating pain scores (VRS, 0–10) or VAS (0–10) were converted to 0–100 mm VAS pain scores for analysis. All opioid analgesics dosages were converted to morphine equivalents (mg) [18]. Data reported in milligram per kilogram were converted to total milligram by multiplying the mean weight of the group. All data were recorded in mean and SD, and data expressed in SE were converted to SD. We extracted the data at the time which was closest to our pre-defined time points of 4, 8, 12, 24, 48 and 72 h. Studies with multiple control groups were used in different comparisons. The number of participants was adjusted to reflect the multiple comparisons.

### Quality assessment

The quality of studies was reviewed using the Cochrane Collaboration’s tool for assessing the risk of bias [12]. Areas of methodologic quality assessed included concealment of allocation (selection bias), random sequence generation (selection bias), blinding of the participants (performance bias), and blinding of outcome assessment (detection bias). We graded each domain as low risk, unclear (uncertain risk) or high risk, according to the criteria outlined in the Cochrane Handbook for Systematic Reviews of Interventions provided by Higgins [17]. Intervention details such as acupuncture rationale, details of needling, treatment regimen, time selection, practitioner background and confidence, and adequacy of stimulation were extracted and evaluated by W.XQ and X.Q according to Standards for Reporting Interventions in Controlled Trials of Acupuncture [19].

### Statistical analysis

Meta-analysis was performed using RevMan 5.2 software. Continuous data was analyzed and presented as mean difference with 95% confidence interval (CI), while dichotomous data was analyzed as RR (relative risk) with 95%CI using random effects model. Forest plots were performed to evaluate the effects. The percentage of heterogeneity was assessed with the I^2^ statistic. I^2^ values of 25, 50, and 75% represent low, moderate and high heterogeneity. Subgroup analysis was conducted if sufficient data were available.

## Results

**Fig. 1:**
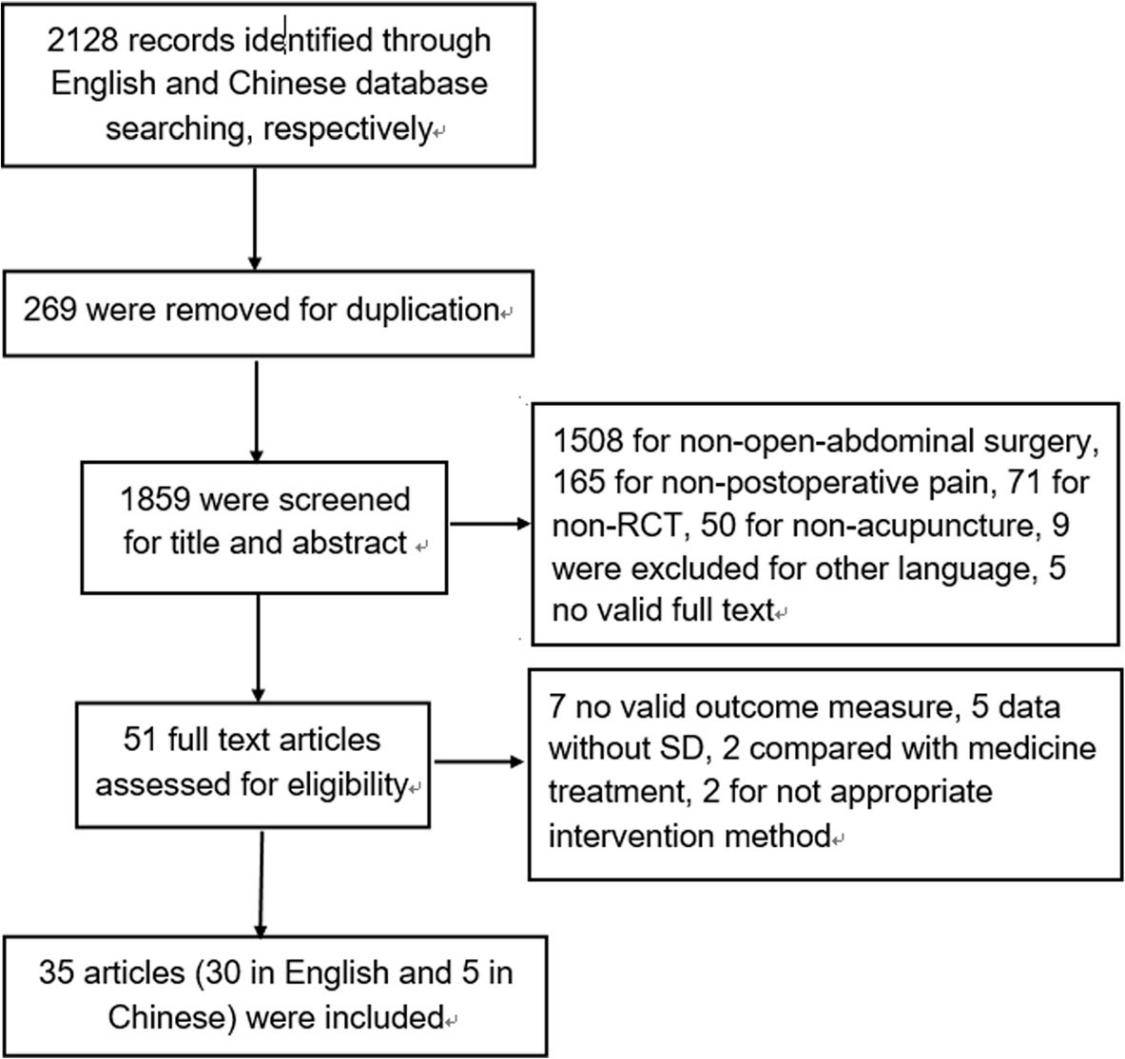
Flow diagram of the search process and study selection

The flow chart of study screening is presented in Fig. 1. The search was first run for the original review in September 2015, updated in September 2016 and we combined both search results. A total of 2128 records were identified, and 269 were removed as duplicates. After screening from the titles and abstracts, 1808 were excluded and 51 potentially eligible studies were left for examination of the full text. Out of 51, 16 were excluded for the reasons outlined in Fig. 1. Finally 35 studies were included in this review, of which 5 were in Chinese and 30 were in English.

### Study characteristics

As shown in Table 1, all participants included had upper or lower abdominal surgeries in this study. Seven studies included caesarean section [20–26], nine studies included gynecological surgery [27–35], three included cholestectomy [36–38], two included appendicectomy [39, 40], three included gastrointestinal surgery [41–43] and 10 studies didn’t mention the types of abdominal surgery. One study included patients undergoing both cesarean resection and vaginal delivery, and we only included data from caesarean section [24].

**Table 1:** Study characteristics of all randomized, controlled trials included in the analysis

Study ID	anesthesia type	Surgery type	Number of patients (A/C)	Stimulation style	Points used in the real acupuncture treatment	Sham/control	Duration	time select
Peri-incisional stimulation								
Binder et al. (2011) [23]	EDA	cesarean section	22/20	Hi-TENS	Incision: 2–3 cm distance from the wound	Non treatment	24 h*1 day	Post-surgery:immediately after surgery, at least 24 h/one day
Bjersa and Andersson(2014) [50]	GA	pancreas resection	9/11	High frequency TENS	Incision:cranial of the incision, sacral of the incision, and columna innervating the dermatome for Th5 to Th9	‘increase stimulation until first sensitivity occurs and keep the stimulation as low as possible	At least 30 min, as much as possible	Post-surgery:two to four hours prior to epidural anlgesia ended
Smith et al. (1986) [30]	GA	caesarean section	9/9	TENS	Incision:the incision site	No electrical current was applied	3 days, with the stimulator being turned off only occasionally, for short intervals (less than 15 min),	Post-surgery:As soon as the patient’s admission to the recovery room
Rakel et al. (2003) [31]	GA	abdominal surgery	32/32	TENS	Incision:parallel to the incision,	No stimulation	No reported	Post-surgery: After surgery,details
Hamza et al. (1999) [32]	GA	gynecologic surgery	25/25	TENS	Incision:surgical incision	No electrical current was applied	30 min, every 2 h	Post-surgery::in the postanesthesia care unit for 30 min at intervals of 2 h or longer
Jaafarpour et al. (2008) [25]	SA	ceserean section	54/54	TENS	Incision: paraspinus muscles at T10-L1, and S2–4. 5 cm above and below the surgical incision,	Non-acu	continuously used for first 24 h except temporary breaks for walking,	Post-surgery: After surgery
Galloway et al. (1984) [36]	GA	Cholecystectomy with or without choledochotomy	14/14	tes	Incision:details	Nonsegmental TES	Not reported	Post-surgery: In the recovery room
Kayman-Kose et al. (2014) [24]	GA	cesarean section	50/50	TENS	Incision:above and below the incision line in women	no electrical current was transmitted in the placebo group	Not reported	Post-surgery: 30 min after childbirth was completed.
Gary et al. (1993) [33]	GA	gynecological operations.	17/13	TENS	Incision:2 cm away from and parallel to the incision	No current applied	24 h, and 20 min on days 2 and 3	Post-surgery: first 24 h after the operation.
Hershman et al. (1989) [37]	GA	cholecystectomy or colorectal surgery	23/22	TENS	Incision:	Non-functioning batteries	Not reported	Post-surgery: until the end of the second post-operative day,
Lim et al. (1983) [51]	GA	upper abdominal surgery	15/15	TENS	Incision:1 cm away from the incision	Reversed batteries	Not reported	Post-surgery: After surgery, the stimulator was turned on in the recovery room
Sodipo et al. (1980) [52]	GA	upper abdominal surgery	15/15	TENS	Incision:2 cm away from the incision	Non-acu	Not reported	Post-surgery: post operation, details
Conn et al.(1986) [39]	GA	Appendectomy	15/13	TENS	Incision:either side of the wound	The machine was switched off	Not reported	Post-surgery: At the end of the procedure
Hargreaves et al. (1989) [53]	Not mentioned	abdominal surgery	25/25	TENS	Incision: NR	No stimulation	Maybe 2 days	Post-surgery:depended on both time of return from surgery and time of dressing change.
Dougal T et al. (1991) [38]	GA	Cholestectomy	15/15	TENS	Incision:each side of incision	Non-acu	Not reported	Post-surgery: in recovery room
CUSCHIERI et al. (1985) [54]	GA	abdominal surgery	53/53	TENS	Incision:bilaterly in the paravertebral area;electroacupuncture:	The batteries are reversed	30 min	Post-surgery: In the recovery room
Bjersa et al. (2015) [43]	EDA	COLON SURGERY	15/13	TENS	Incision:bilaterally of the incision,and columna innervating Th10 to L1	“increase stimulation until first sensitivity occurs and keep the stimulation as low as possible”	no time limitations	Post-surgery: 1 h prior to EDA termination
Distal acupoint stimulation								
Hui et al. (2002) [20]	EDA	caesarean section	20/20	EA	Distal:Zusanli, Sanyinjiao	Non-acu	30 min	Combined: pre-operation and 4 h or 8 h after surgery
Baoguo et al. (1997) [44]	GA	lower abdominal surgery	25/25	EA	Distal:hegu	No electrical current was applied	30 min, every 2 h	Post-surgery::After surgery,details
He et al. (2007) [41]	GA	radical operation of intestinal cancer	30/30	EA	Distal:scalp	Non-acu	until the end of surgery	Pre-surgery: 20 min before surgery
Chen et al. (1998) [27]	GA	hysterectomy or myomectomy	25/25	EA	Distal:Zusanli	Nonacupoint and no-current	every 2–3 h	Post-surgery:On arrival in the PACU,
Lin et al.(2002) [45]	GA	lower abdominal surgery	25/25	EA	Distal:Zusanli,	with the indicator light on but with no actual current	20 min	Pre-surgery: 20 min prior to anesthesia.
Feng et al.(2013) [28]	EDA	Gynecological surgery	20/20	EA	Distal:Zusanli, Sanyinjiao	Non-acu	Not reported	Pre-surgery:details
Feng et al.(2010) [29]	EDA	Gynecological surgery	20/20	EA	Distal:Zusanli, Sanyinjiao	Non-acu	Not reported	Pre-surgery: Before surgery
Adib-Hajbaghery et al. (2013) [40]	GA	appendectomy	35/35	Acupressure	Distal:Le7	the button placed right in opposite side of the Le7 point (as sham point)	1 h	Post-surgery: After surgery, started after the full patient’s consciousness
Kim et al.(2006) [35]	GA	Abdominal hysterectomy	30/30	Capsicum Plaster	Distal:Zusanli	inactive tape (5 _ 5 mm2) without PAS was applied to the Zusanli point of both legs.	8 h per day/3 days	Combined: before induction of anesthesia
Hsiung et al.(2015) [42]	GA	subtotal gastrectomy	26/28	Acupressure	Distal:Neiguan (P6) and Zusanli (ST36)	Non-acu	Three12-min	Post-surgery:consecutive days following surgery.
Ntritsou et al. (2014) [46]	GA	radical prostatectomy	35/35	EA	Distal:LI4,ST36	No current was applied	30 min	Post-surgery: when the closure of the abdominal walls was initiated
Lee et al.(2011) [47]	GA	hysterectomy	13/12	EA	Distal:Zusanli (ST36)	no stimulation	30 min	combined: before general anesthesia, and initiated once regained consciousness
Kotani et al.(2001) [48]	GA	elective upper and lower abdominal surgery	50/48	Acupuncture	Distal:BL18 –BL24 or BL20 –BL26	a needle was positioned at each acupoint	4 days	Combined: Before induction of anesthesia,
Wu et al. (2009)	SA	cesarean section	20/20	EA	Distal:Sanyinjiao(Sp6)	Non-acu	30 min	Post-surgery: In the recovery room
Li et al. (2012) [22]	CESA	cesarean section	60/60	EA	Distal:auricular Shenmen point(TF4)	No current applied	30 min	Combined: before surgery and 3 h, 8 h after surgery
Masatomo et al. (2003) [49]	GA	abdominal surgery.	23/30	Acupressure	Distal:neiguan, Zusanli, Sanyinjiao, and Gongsunpoints	Non-acu	Not	Post-surgery: On completion of surgery
Tsang et al.(2011) [34]	GA	total abdominal hysterectomy	16/16	auricular acupuncture	Distal: “uterus”“abdomen”“sympathetic” “shenmen” and“subcortex”	The sham TENS group received auricular TENS delivered to the following five inappropriate points: “teeth,” “tongue,” “mandible,” “eye,” and “face”	20 min	Post-surgery: after surgery,
Combined distal and peri-incisional stimulation								
Sim et al. (2002) [26]	GA	gynaecologic lower abdominal surgery	30/30	EA	Distal:ST36 and PC6 bilaterally and subcutaneously along the skin incision.	No electrical current was applied.	45 min	Post-surgery:group1\2:45 min before induction of anesthesia (GA)Group 3–45 min of postoperative

*CESA* combined epidural and spinal anesthesia**,** *EA* electroacupuncture**,** *EDA* epidural anesthesia**,** *GA* general anesthesia**,** *NR* not reported**,** *SA* spinal anesthesia**,** *TENS* Transcutaneous Electrical Nerve Stimulation

Twenty-six studies applied general anesthesia (76.5%), eight studies used spinal anesthesia or epidural anesthesia or combined spinal and epidural anesthesia, and one study did not mention the anesthesia type. The age and gender were comparable between intervention and control groups in all studies, and all trials were for adults.

#### STRICTA

Seventeen trials used distal acupoint stimulation [20–22, 27–29, 34, 35, 40–42, 44–49] and 17 trials used peri-incisional stimulation [23–25, 30–33, 36–39, 43, 50–54], and one trial combined distal and peri-incisional stimulation [26].

For distal stimulation, six types of stimulation were included: electroacupuncture (EA) [20–22, 27–29, 41, 45–47], transcutaneous acupoint electric stimulation(TEAS) [44], manual acupuncture [48], acupressure [40, 42, 49], auricular acupuncture [34] and capsicum plaster [35]. For peri-incisional stimulation, all studies stimulated at the peri-incisional area using the TENS or TENS like device.

#### Adequacy of distal acupuncture or peri-incisional treatment protocol

All studies described the details of treatment. None of the distal acupuncture studies provided a diagnosis according to Chinese medicine. Only two out of 17 distal studies gave references and literatures for their decision on acupuncture points selection [34, 44]. Most studies in peri-incisional group described the site as “around or 1 to 3cm away from skin incision”.

For distal acupuncture, seven acupoints were used, including Zusanli (ST36), Sanyinjiao (SP6), Neiguan (P6), Hegu (LI4), Gongsun(SP4), Shenmen (TF4), and Lanwei (Le7). ST36 and SP6 on the legs were two of the most frequently used distal acupoints, and used in eleven and six studies, respectively. Most of the studies used bilateral needling, except for seven studies [24, 33, 36, 37, 40, 51, 53] which did not state unilateral/bilateral needling details. Nine studies clearly stated how many needles were inserted, varying from 2 to 14, other studies didn’t offer the needle numbers.

With respect to the depth of needle insertion, three studies clearly described a depth of 0.2 cm for LI4 and 3-4 cm and 0.5-1 cm at acupoints ST36 and PC6, and subcutaneously for auricular acupuncture respectively [26, 46]. Other trials did not mention the depth of needle. Most studies stimulated at the highest intensity that patients could tolerate, while two studies in distal group described the intensity of “deqi” [42, 45]. This is not relevant to those peri-incisional stimulation where all of them use surface electrodes. The frequency and intensity of stimulation and retention time ranged widely among studies and were well reported in most studies.

Stimulation was initiated before surgery and after surgery in 11 studies and 24 studies respectively. Out of 11 studies that initiated before surgery, four studies maintained stimulating through the surgery [33, 35, 41, 48], three repeated acupuncture daily or every a few hours after surgery [20, 22, 47], two with no further stimulation [29, 45], while another two studies did not report details. No trial employed only intraoperative acupuncture. Out of the 24 studies that initiated after surgery, eight studies stimulated shortly from 12 min to 1 h after surgery, five studies stimulated daily during day 1 to day 4 postoperatively, and others didn’t describe the details.

#### Comparison groups

Of the 17 distal stimulation studies, five studies compared with sham treatment [27, 35, 40, 46, 48], seven studies compared with non-active treatment [20, 21, 28, 29, 41, 42, 49] and five studies compared with both forms of controls [22, 34, 44, 45, 47]. Two out of 17 studies compared different frequencies of stimulation [45, 47], and 1/17 compared EA with manual acupuncture [21]. In the 17 peri-incisional stimulation studies, 11 compared with sham [24, 30, 32, 33, 36–38, 43, 50, 51, 54], three compared with non-active stimulation [23, 25, 52], and three compared with both [31, 39, 53]. In addition, one study compared different intensities of stimulation [30], and one compared different frequencies of stimulation [32]. The control group of the combined distal and peri-incisional study was sham stimulation [26].

For the sham treatment, two studies (Bjersa 2014 and 2015) applied a low pulse intensity stimulus in their comparison group as the researchers felt this was more credible than a no stimulus placebo. Three distal studies [27, 34, 40] and one peri-incisional study [36] applied the stimulation to nonacupoint or inappropriate acupoints as the placebo control. The other 20 studies used a sham device that was similar to the active device without electrical current.

### Risk of bias in included studies

**Fig. 2:**
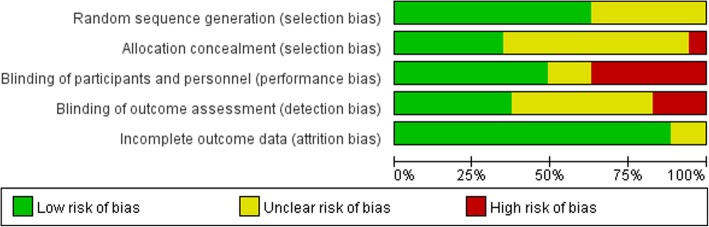
Risk of bias graph: review authors’ judgements about each risk of bias item presented as percentages across included studies

A Risk of bias graph of each study is presented in Fig. 2. Four out of 35 studies were with an overall low risk of bias, and were studies of distal acupuncture. Fourteen studies were considered with a high risk of bias as one or more key domains were rated as ‘high risk’. One or more “unclear risks” were included in remaining 17 studies.

#### Randomization and allocation concealment

Allocation sequence generation was rated as “low risk” in 23 studies, which was produced using a computer-generated random numbers table [21, 31, 34, 35, 42, 44–46, 48, 49], by an independent person blinded to the study design [23, 43, 50], a table of random numbers [22, 24, 28, 29, 33, 39, 41, 47] and a block design technique [36, 37].

Twelve of the 35 trials described allocation concealment [23, 24, 34, 35, 37, 39, 42, 43, 46–48, 50] and rated as “low risk”, the remaining trials did not report clear allocation concealment and were rated as “unclear”.

#### Blinding

Operators who delivered stimulations could not be blinded to the allocated treatment, while participants could be blinded by using placebo or sham control. Participants in all 11 studies that compared non-acupuncture with acupuncture treatment were not blinded, and were rated as “high risk”. In 21 trials, the blinding of outcome assessors was unclear, therefore performance bias was likely to have occurred.

#### Incomplete outcome data

All trials reported the complete outcome data, so attrition bias was low.

### Outcomes

#### Primary outcomes

##### Distal acupuncture or Peri-incisional stimulation versus controls Intensity of postoperative pain at rest

**Fig. 3:**
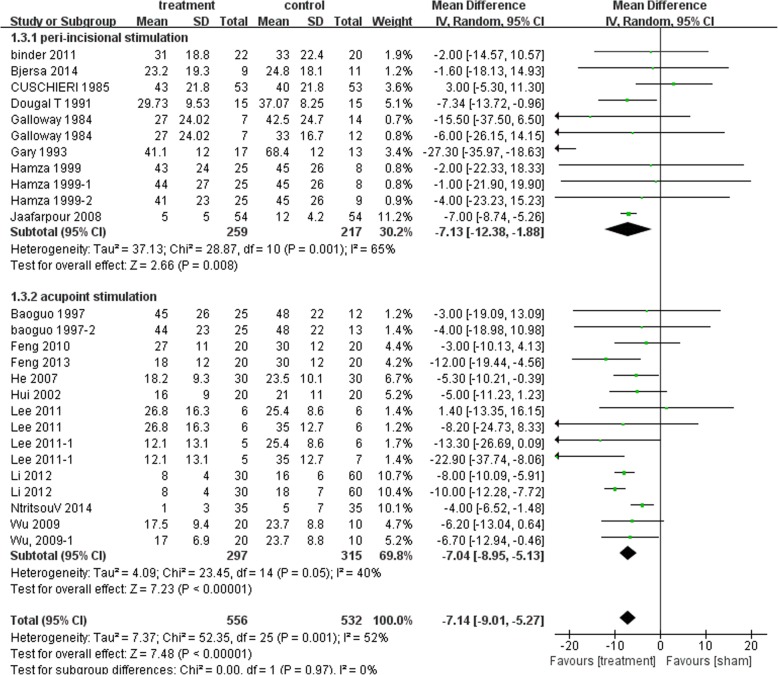
Postoperative resting pain intensity at 24 h: Subgroup analysis of Peri-incisional stimulation vs distal stimulation

Postoperative pain at 4 h, 12 h, 24 h and 48 h was available for this comparison. Distal acupuncture was found significantly more effective than their controls in reducing postoperative pain intensity at rest at different time points[4 h: MD − 11.82 mm, 95% (− 15.47, − 8.16), I^2^ 64%; 12 h: MD − 11.92 mm, 95%CI (− 13.58, − 10.26), I^2^ 84%; 24 h: MD − 7.14 mm, 95%CI (− 8.95, − 5.13), I^2^ 40%; 48 h: MD − 9.45 mm, 95%CI (− 12.41, − 6.50), I^2^ 68%]. Peri-incisional stimulation also showed beneficial effects compared with their controls. [4 h: MD − 10.70 mm, 95% CI (− 15.32,-6.0), I^2^ 45%; 12 h: MD − 13.52 mm, 95% CI (− 15.25, − 11.78), I^2^ 92%; 24 h: MD − 7.13 mm, 95%CI (− 12.38, − 1.88), I^2^ 65%; 48 h: − 10.32 mm, 95% CI (− 14.28, − 6.37), I^2^ 47%]. Figure 3 showed the 24 h comparison data for postoperative pain at rest.

#### Intensity of postoperative pain on movement or cough

**Fig. 4:**
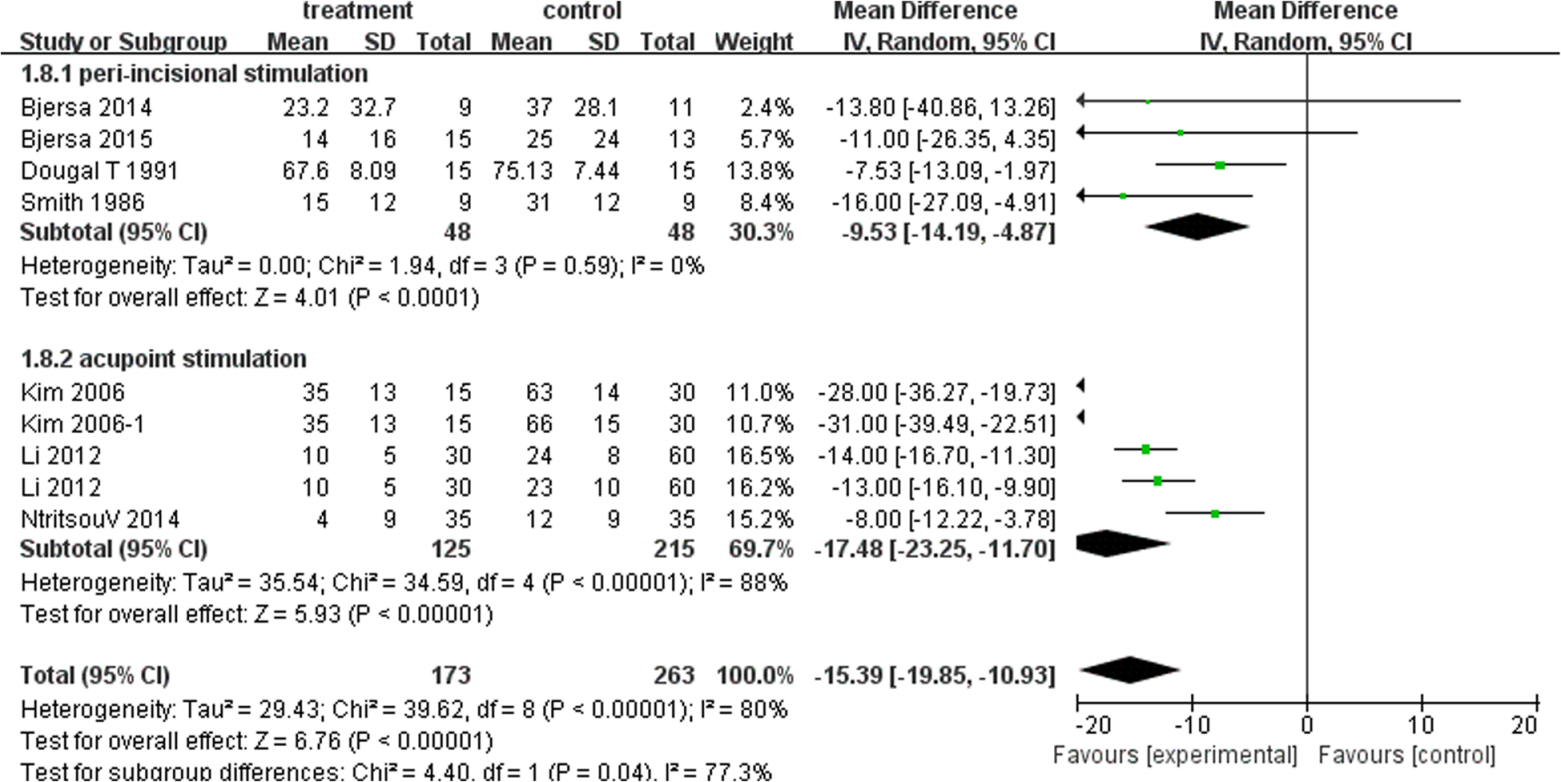
Postoperative pain intensity on movement at 24 h: Subgroup analysis of Peri-incisional stimulation vs distal stimulation

Eight studies had suitable data for this comparison. For postoperative pain on movement, distal acupuncture showed better effects than controls at 4 h [MD − 26.49 mm, 95% CI (− 35.56, − 17.42), I^2^ 83%], 24 h [distal: − 17.48 mm, 95%CI (− 23.25, − 11.70), I^2^ 88%] and 48 h [distal: − 16.61 mm, 95%CI (− 21.95, − 11.62), I^2^ 82%]. Peri-incisional stimulation also showed beneficial effects compared with their controls at 4 h [MD − 4.46 mm, 95% CI (− 13.62, 4.70), I^2^ 0%], 24 h [;-9.53 mm, 95% CI(− 14.19, − 4.87), I^2^ 0%] and 48 h [− 14.02 mm, 95%CI (− 19.06, − 8.98), I^2^ 0%]. Figure 4 showed the 24 h comparison data.

#### Postoperative opioid consumption

**Fig. 5:**
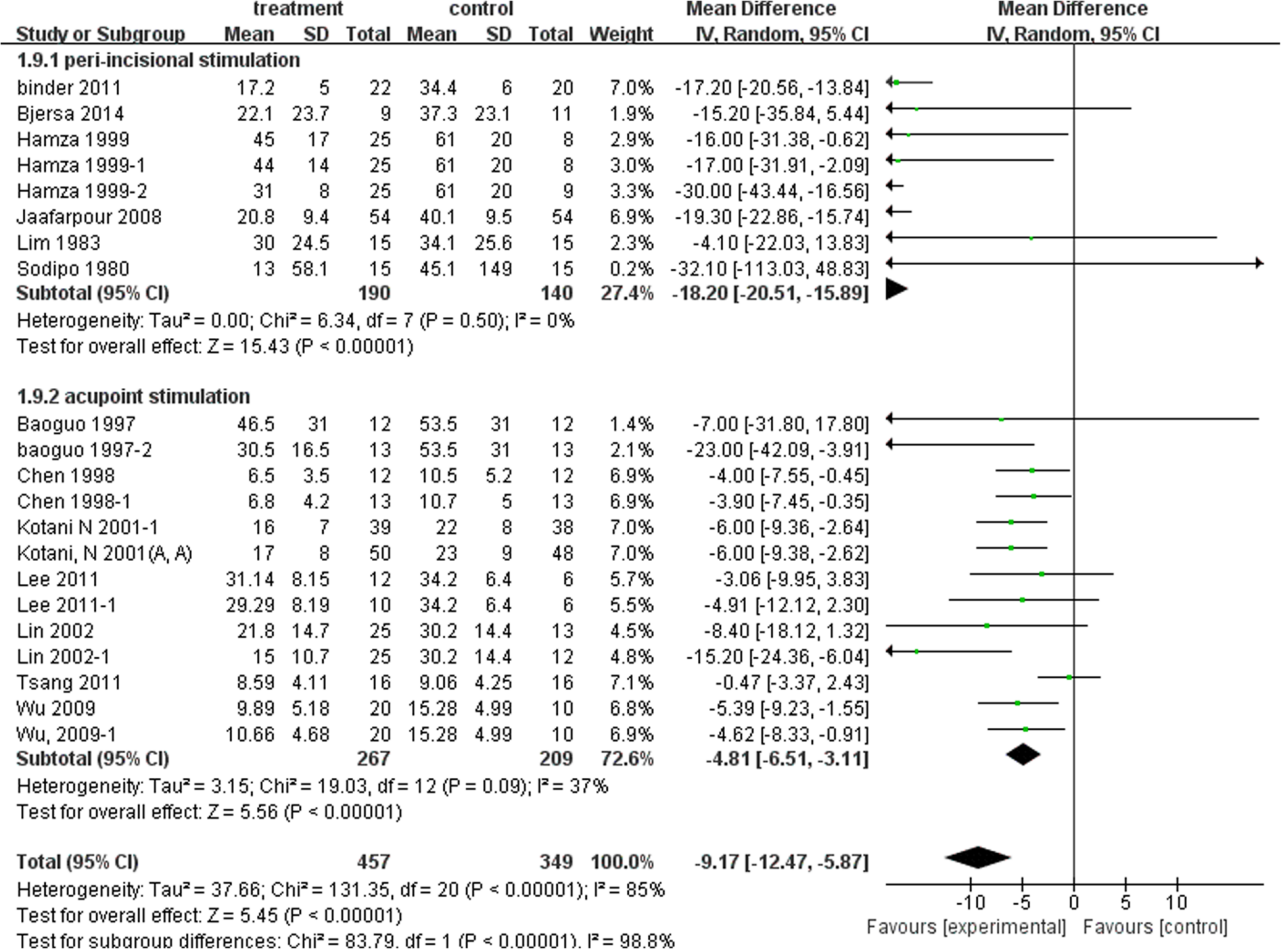
Postoperative opioid consumption at 24 h: Subgroup analysis of Peri-incisional stimulation vs distal stimulation

In Fig. 5, both distal acupuncture and peri-incisional stimulation showed significant reduction of postoperative opioid consumption at 24 h [distal: MD − 4.81 mg, 95%CI (− 6.51, − 3.11), I^2^ 37%; Peri-incisional: MD − 18.2 mg, 95% CI (− 20.51, − 15.89), I^2^ 0%].

##### Distal acupuncture versus Peri-incisional stimulation

No studies directly compared the two modes of stimulation. Fifteen studies in incision group and 20 studies in distal group had suitable data for sub-group comparisons. Subgroup analysis showed no significant difference between the effects of peri-incisional points and distal stimulation in alleviating postoperative resting pain at any time point (*P* = 0.17, 0.19, 1.0 and 0.06 at 4 h, 12 h, 24 h and 48 h respectively).

Postoperative cough pain at 24 h and 48 h showed no difference between two modes of stimulation. However, the distal acupuncture group had statistically significantly less pain on movement or cough at 4 h compared with incision group (Chi^2^ = 20.35, *P* < 0.00001).

Cumulative opioid consumption at 24 h postoperatively was significantly lower in incision group when compared with that in distal group (Chi^2^ = 88.82, *P* < 0.00001).

##### Peri-incisional or distal versus combination of both

No studies compared the combined of the two modes with either distal or peri-incisional stimulation alone. Only one study compared combination of the two stimulation modes with sham stimulation, and showed that preoperative stimulation could reduce 24 h morphine consumption. Subgroup analysis was not available.

#### Secondary outcomes

##### Opioid related adverse events

Seven studies in distal acupuncture group reported opioid related adverse events, while no study in peri-incisional group reported. The incidence of nausea significantly decreased in the distal acupuncture group compared with sham treatment (RR 0.7, 95%CI 0.55, 0.91, I^2^ = 39%), while vomiting was reduced without statistically significant difference (RR 0.64, 95%CI 0.36, 1.04, I^2^ = 0%). Four studies reported that acupuncture was associated with a lower incidence of postoperative dizziness (RR 0.67, 95%CI 0.51, 0.88, I^2^ = 18%), while it was not better than controls in the incidence of pruritis (RR 0.72, 95%CI 0.42, 1.23, I^2^ = 0%).

##### Distal or peri-incisional stimulation related side-effects

Three studies in distal group and one study in peri-incisional group reported side effects associated with the treatment [32, 35, 50, 52]. Side effects included erythema due to Capsicum plaster, a tingling and transient warm sensation, restricting activities and discomfort influencing sleep quality due to electrodes and wires. All of these side-effects were resolved spontaneously and were comparable with the control group. Seven studies reported no adverse effects or uncomfortable sensation. Others didn’t report acupuncture- related adverse effects.

## Discussion

### Summary of findings

In this meta-analysis, we found that distal acupoint stimulation and peri-incisional stimulation both had positive effects on reducing postoperative resting pain as well as pain on movement or cough up to 72 h postoperatively when compared with sham or non-treatment controls. In addition, both reduced postoperative opioid consumption at 24 h. Subgroup analysis showed no difference between peri-incisional or distal stimulation on postoperative pain reduction. However, peri-incisional stimulation was superior in reducing opioid consumption at 24 h whereas distal acupoint stimulation reduced opioid-related adverse effects, including nausea and dizziness. The pain intensity on movement at postoperative 4 h was lower in distal stimulation.

The level of evidence is low to moderate due to a moderate to high heterogeneity among studies and about half of the included trials had less than 25 participants in each study arm. The degree of risk of biases across trials also varied, with only four studies rated at overall low risks.

### Strengths and limitations

There are several strengths of this review. First, we employed a comprehensive search of the Chinese and English databases with no language restriction. Secondly, we studied postoperative pain, both at rest and movement evoked, which is highly relevant to clinical practice. Thirdly, we restricted our analysis to studies on open abdominal surgery to reduce the heterogeneity introduced by different surgery types.

The current review also has several limitations. Firstly, we included trials using various stimulation techniques, manual, EA, acupressure and auricular acupuncture in distal acupuncture group. Sensitivity analysis was conducted by restricting stimulation to EA only or to English trials only and found this broad inclusion did not impact on the results of the review. Secondly, the heterogeneity was high in most comparisons, and we used a random effect model to address it. In addition, the broad difference of treatment duration and frequency among studies may contribute to the high heterogeneity. Thirdly, the number of participants randomized to each treatment group ranged from 9 to 60, with half of them having less than 25 participants in each study arm, which is a small sample size for pain studies [55]. Most of the studies did not justify the sample size calculation. Due to those limitations, we have downgraded the level of evidence and interpreted the data with caution.

### Clinical relevance of distal acupuncture and peri-incisional stimulation

The site of delivering non-drug stimulation is highly relevant to clinical practice. To our knowledge, this is the first systematic review that examines the difference between incision and distal stimulation on postoperative pain and opioid sparing.

Peri-incisional stimulation is easy to use, noninvasive and often applied continuously for 24 h to 4 days after surgery, but the concerns of infecting the incision site are high. On the contrary, distal acupuncture is applied to arms and legs and could be applied before or after surgery and repeated every day for a short time without influencing the incision site, which is around abdomen in this review. Distal acupuncture could also be non-invasive. In one of the included studies, surface electrodes were applied to acupoints, i.e., TEAS. For the types of distal acupuncture, the review by Wu and colleagues (2016) found that conventional manual acupuncture and TEAS were associated with less postoperative pain than control treatment, while EA was similar to control; TEAS on acupoints was associated with significantly greater reduction in opioid analgesics use than control while conventional acupuncture and EA showed no benefit when compared with controls [56]. TEAS on distal acupoints may be an alternative in treating postoperative pain.

Either distal or peri-incisional stimulation was effective, however the later was more effective in opioid sparing. So the choice of which mode of stimulation will depend on the magnitude of the concerns over local infection and the importance of opioid sparing. Due to a lack of studies, we could not comment on whether a combination of peri-incisional and distal point stimulation is better than either alone.

### Outcome data relevant to knowledge translation

To enable evidence being translated into practice, we have examined parameters that are essential to clinical practice in this review, such as the types of pain and safety data.

In recent years, increasing attention has been placed on movement-evoked pain as it is closely associated with postoperative thromboembolic complications and pulmonary dysfunction [57]. In addition, pain on movement adversely influences patient ambulation and early recovery in the early postoperative period [58, 59], and opioids were found relatively ineffective in treating pain on movement [58]. It is essential to study the effects of acupuncture on pain at rest as well pain on movement. In our review, only 9 studies (25.7%) included pain on movement as an outcome measure. Distal and peri-incisional stimulation was equally effective in pain on movement with the former being more effective at the early stage postoperatively than peri-incisional stimulation. Both present themselves as excellent adjunct non-pharmacological therapies in multimodal analgesic strategies.

Three studies in distal group and one study in peri-incisional group reported transient and minor adverse events, which is consistent with Sun’s review. A prospective survey studied the adverse effects of acupuncture and reported a rate of 14 per 10,000, and the events were minor [60]. Another survey also showed minor events after 34,407 acupuncture treatments [61]. Safety data from primary care cannot however be readily translated to the safety of acupuncture in acute and sub-acute settings. Peri-operative distal acupuncture or peri-incisional stimulation is likely to be safe, but future studies should report detailed safety data.

### Implications for future studies

Considering the severe adverse events associated with current drug therapies for postoperative pain, the use of non-pharmacological interventions should be encouraged. Future studies could investigate the combination effects of distal and peri-incisional stimulation and directly compare the effects of distal and peri-incisional stimulation. Studies should report both resting pain and movement pain at different time points postoperatively. Factors that are related to evidence translation should be investigated, such as patients’ preferences and feasibility of integrating those therapies in to routine management of postoperative pain.

## Conclusion

Perioperative distal acupoint or peri-incisional stimulation is safe and effective for postoperative pain and opioid sparing. They could be alternative or adjunct analgesic intervention. Which forms of stimulation to be used depends on the needs and availability of instruments and personnel. Distal acupuncture could be more effective in reducing movement pain at the early stage of post surgery, whereas peri-incisional stimulation was more effective in reducing postoperative opioids use. More studies with a large sample size that directly compare the two forms of stimulation are needed in the future.

## Abbreviations

CENTRAL: Cochrane central register of controlled trials
EA: Electroacupuncture
RCTs: Randomized control trials
RR: Relative risk
TEAS: Transcutaneous acupoint electric stimulation
TENS: Transcutaneous electrical nerve stimulation
